# Craniofacial bone anomalies related to cholesterol synthesis defects

**DOI:** 10.1038/s41598-024-55998-3

**Published:** 2024-03-04

**Authors:** Chihiro Iwaya, Akiko Suzuki, Junbo Shim, Aemin Kim, Junichi Iwata

**Affiliations:** 1 Department of Diagnostic and Biomedical Sciences, The University of Texas Health Science Center at Houston (UTHealth), School of Dentistry, 1941 East Road, BBS 4208, Houston, TX 77054 USA; 2 Center for Craniofacial Research, The University of Texas Health Science Center at Houston, School of Dentistry, Houston, TX 77054 USA; 3 MD Anderson Cancer Center UTHealth Graduate School of Biomedical Sciences, Houston, TX 77030 USA; 4 Present Address: University of Missouri - Kansas City, School of Dentistry, Kansas City, MO 64108 USA

**Keywords:** Bone development, Disease model

## Abstract

DHCR7 and SC5D are enzymes crucial for cholesterol biosynthesis, and mutations in their genes are associated with developmental disorders, which are characterized by craniofacial deformities. We have recently reported that a loss of either *Dhcr7* or *Sc5d* results in a failure in osteoblast differentiation. However, it remains unclear to what extent a loss of function in either DHCR7 or SC5D affects craniofacial skeletal formation. Here, using micro computed tomography (μCT), we found that the bone phenotype differs in *Dhcr7*^*−/−*^ and *Sc5d*^*−/−*^ mice in a location-specific fashion. For instance, in *Sc5d*^*−/−*^ mice, although craniofacial bones were overall affected, some bone segments, such as the anterior part of the premaxilla, the anterior–posterior length of the frontal bone, and the main body of the mandible, did not present significant differences compared to WT controls. By contrast, in *Dhcr7*^*−/−*^ mice, while craniofacial bones were not much affected, the frontal bone was larger in width and volume, and the maxilla and palatine bone were hypoplastic, compared to WT controls. Interestingly the mandible in *Dhcr7*^*−/−*^ mice was mainly affected at the condylar region, not the body. Thus, these results help us understand which bones and how greatly they are affected by cholesterol metabolism aberrations in *Dhcr7*^*−/−*^ and *Sc5d*^*−/−*^ mice.

## Introduction

Cholesterol is a source of bile acids, steroid hormones, and oxysterols in the body and is a vital component of cellular membranes^1^. Cholesterol biosynthesis is regulated through multiple, highly coordinated steps, which involve over 30 reactions catalyzed by more than 15 enzymes^2^. Sterol-C5-desaturase (SC5D) converts cholesta-7,24-dien-3β-ol and lathosterol into 7-dehydrodesmosterol (7-DHD) and 7-dehydrocholesterol (7-DHC), whereas 7-dehydrocholesterol reductase (DHCR7) catalyzes 7-DHD and 7-DHC to desmosterol and cholesterol.

Recent studies indicate that dysregulation of cholesterol synthesis is associated with various bone disorders and diseases^3^. In humans, mutations in *SC5D* cause lathosterolosis, which is characterized by growth retardation and intellectual disability, short limbs, polydactyly/syndactyly, and craniofacial malformations including cleft palate, micrognathia, midfacial hypoplasia, and calvarial defects^4^. Mutations in *DHCR7* cause Smith-Lemli-Opitz syndrome (SLOS), with cleft palate, postaxial polydactyly, 2–3 toe syndactyly, microcephaly, micrognathia, and intellectual disability as manifestations in humans^5,6^. Dysfunction of SC5D and DHCR7 leads to lathosterolosis and desmosterolosis, respectively^7–11^. Thus, these clinical manifestations and biochemical features suggest that the inborn errors associated with cholesterol biosynthesis are mainly due to the accumulation of cholesterol precursors, and not due to lower mature cholesterol levels.

As seen in humans, mice deficient for *Sc5d* and *Dhcr7* display defects in bone formation^1,3^. Specifically, mice deficient for *Sc5d* (*Sc5d*^*−/−*^ mice; hereafter *Sc5d* KO mice) exhibit cleft palate, micrognathia, agenesis of the lower incisors, calvaria hypomineralization (defects in intramembranous ossification), malformations in the long bones (defects in endochondral ossification), and syndactyly/polydactyly^4,12^, whereas mice deficient for *Dhcr7* (*Dhcr7*^*−/−*^ mice; hereafter *Dhcr7* KO mice) exhibit accelerated calvarial bone formation and cleft palate, but only in 9% of the mutant mice^13,14^. Moreover, although increased levels of cholesterol precursors and lower levels of mature cholesterol in serum and tissues are commonly detected in newborn *Sc5d* KO and *Dhcr7* KO mice^4,15,16^, these mouse models show different protein expression profiling in the brain^17^. In addition, *Sc5d* KO and *Dhcr7* KO mice display craniofacial skeletal anomalies due to altered hedgehog and WNT/β-catenin signaling pathways at different extents and locations^14,18^. These results suggest that the phenotypic differences between *Sc5d* KO and *Dhcr7* KO mice may be due to the accumulation of different cholesterol precursors.

Previous studies showed that cholesterol metabolism aberrations lead to craniofacial bone anomalies^3^; however, it remains unclear how an aberrant accumulation of cholesterol intermediates or loss of mature cholesterol specifically affects bone morphology. In this study, we investigated how the accumulated cholesterol precursors affect bone size in mouse models with a deficiency in cholesterol biosynthesis. Comparing the size of craniofacial bones across these mouse models allows us to identify specific areas that are affected during craniofacial development. To determine the contributions of ectopic accumulation of different cholesterol intermediates, as well as of the absence of mature cholesterol, to the pathogenesis of these diseases, we analyzed bone morphology in *Sc5d* KO and *Dhcr7* KO mice with high-resolution μCT, and measured length and volume using 3D-reconstructed images.

## Materials and methods

### Animals

*Sc5d*^+/-^ and *Dhcr7*^+*/−*^ mice were a gift from Dr. Forbes D. Porter (The Eunice Kennedy Shriver National Institute of Child Health and Human Development, National Institutes of Health, Bethesda, Maryland, USA). Genotyping was performed using PCR primers as previously described^4,14^. All mice were bred under pathogen-free conditions, with free access to water and food and a 12-h light/dark cycle, in the UTHealth animal facility. All animal experiments were reviewed and approved by the Animal Welfare Committee (AWC) and the Institutional Animal Care and Use Committee of UTHealth (AWC-22–0087). All methods were performed in accordance with the relevant guidelines and regulations provided by ARRIVE (Animal Research: Reporting of In vivo Experiments).

### μCT scanning and three-dimensional (3D) reconstruction

Pregnant mice were euthanized through carbon dioxide (CO_2_) inhalation, and embryos were euthanized through CO_2_ inhalation followed by decapitation, according to the American Veterinary Medical Association (AVMA) Guidelines for the Euthanasia of Animals. Embryos were collected at embryonic day (E) E18.5, fixed with 4% paraformaldehyde overnight, and stored in cold phosphate-buffered saline (PBS) until μCT scanning (n = 6 each genotype). The embryos were placed in a 12-mm diameter sample holder and stabilized with polypropylene straws during scanning. The μCT scans were performed at a 12-µm resolution using the SCANCO µCT-40 system (SCANCO medical USA Inc., USA; 55 kVp and 145 µA); 70% ethanol was used as the scan medium. The scanned images were analyzed using 3D-reconstructed μCT images generated with the Dragonfly software [Version 2021.1 for Windows. Object Research Systems (ORS) Inc., Montreal, Canada] with DICOM files. Craniofacial bones were isolated and labeled using the Dragonfly’s semiautomatic segmentation editor^19,20^. The square root of the sum of the squared distances from the centroid of the landmark configuration to each landmark was quantified as the deviation of the landmark configuration of each specimen. Basic morphometric data were analyzed with principal component analysis (PCA). The distribution of each landmark in mutants (*Sc5d* KO and *Dhcr7* KO) and control littermates (*Sc5d* WT and *Dhcr7* WT) along with each principal component (PC) axis was explained by PC1 and PC2. Landmarks were identified as previously described^19,20^ and are provided in Table 1. We used one of the landmarks as a fixed point in each bone to superimpose the images.

**Table 1:** Selected landmarks of craniofacial bones.

Premaxilla	
1	Most anterior superior point of the premaxilla
2	Most lateral point of the premaxillary-maxillary suture
3	Tip of the frontal process of the premaxilla
4	Most medial point of the premaxillary-maxillary suture
5	Most anterior point of the anterior palatine foramen
6	Most posterior point of the premaxilla
7	Most posterior point of the incisive foramen
Maxilla	
1	Anterior point of the maxilla
2	Lateral inferior intersection of the frontal and zygomatic process of the maxilla
3	Tip of the zygomatic process of the maxilla
4	Anterior-medial point to the zygomatic process of the maxilla
5	Posterior point of the maxilla
6	Posterior-lateral point of the palatine process of the maxilla
7	Posterior-medial point of the palatine process of the maxilla
8	Most anterior-medial point of the palatine process of the maxilla
9	Anterior-lateral point of the palatine process of the maxilla
10	Medial point of the premaxillary-maxillary suture
Palatine bone	
1	Most anterior-lateral point of the palatine plate
2	Tip of the orbital process of the palatine bone
3	Lateral point of the palatine bone
4	Posterior point of the palatine bone
5	Posterior-medial point of the horizontal plate of the palatine bone
6	Anterior-medial point of the horizontal plate of the palatine bone
7	Anterior–superior point of the perpendicular plate
Frontal bone	
1	Most anterior–superior point of the frontal bone
2	Most posterior-superior point of the frontal bone
3	Most posterior-lateral intersection of the frontal bone and parietal bone
4	Most posterior-inferior point of the frontal bone
5	Most anterior-inferior point of the frontal bone
6	Midpoint of the interfrontal suture
Mandible	
1	Most anterior point of the mandible
2	Anterior–superior point of the mandible
3	Mental foramen
4	Molar alveolus of dentary
5	Anterior junction of the mandibular ramus and body
6	Superior tip of the coronary process of the mandible
7	Most inferior point of the mandibular notch
8	Anterior point of the condylar process of the mandible
9	Posterior point of the condylar process of the mandible
10	Superior point of the angular process of the mandible
11	Secondary cartilage of the angular process of the mandible
12	Inferior junction of the mandibular ramus and body
13	Midpoint of the external oblique ridge
14	Inferior point of the mandibular body
15	Mandibular foramen

### Statistical analysis

All results obtained were analyzed with the Prism software (GraphPad Software, California, USA). The statistical significance for multiple pairs of groups was evaluated using a two-way or one-way ANOVA with Tukey’s multiple comparison test. An adjusted *p* < 0.05 was statistically significant. Data are represented as dots and mean ± standard deviation (SD) in the graphs and tables. A multiple variable PCA was conducted using a standardized model, and the PCs were selected based on the percent of total explained variance.

## Results

Throughout this study, we analyzed littermate wild-type (WT) mice as controls in each *Dhcr7* and *Sc5d* strain. In addition, we confirmed that there was no significant difference between WT mice of the *Dhcr7* and *Sc5d* strains (Tables S1-S3).

### Premaxilla

**Figure 1:**
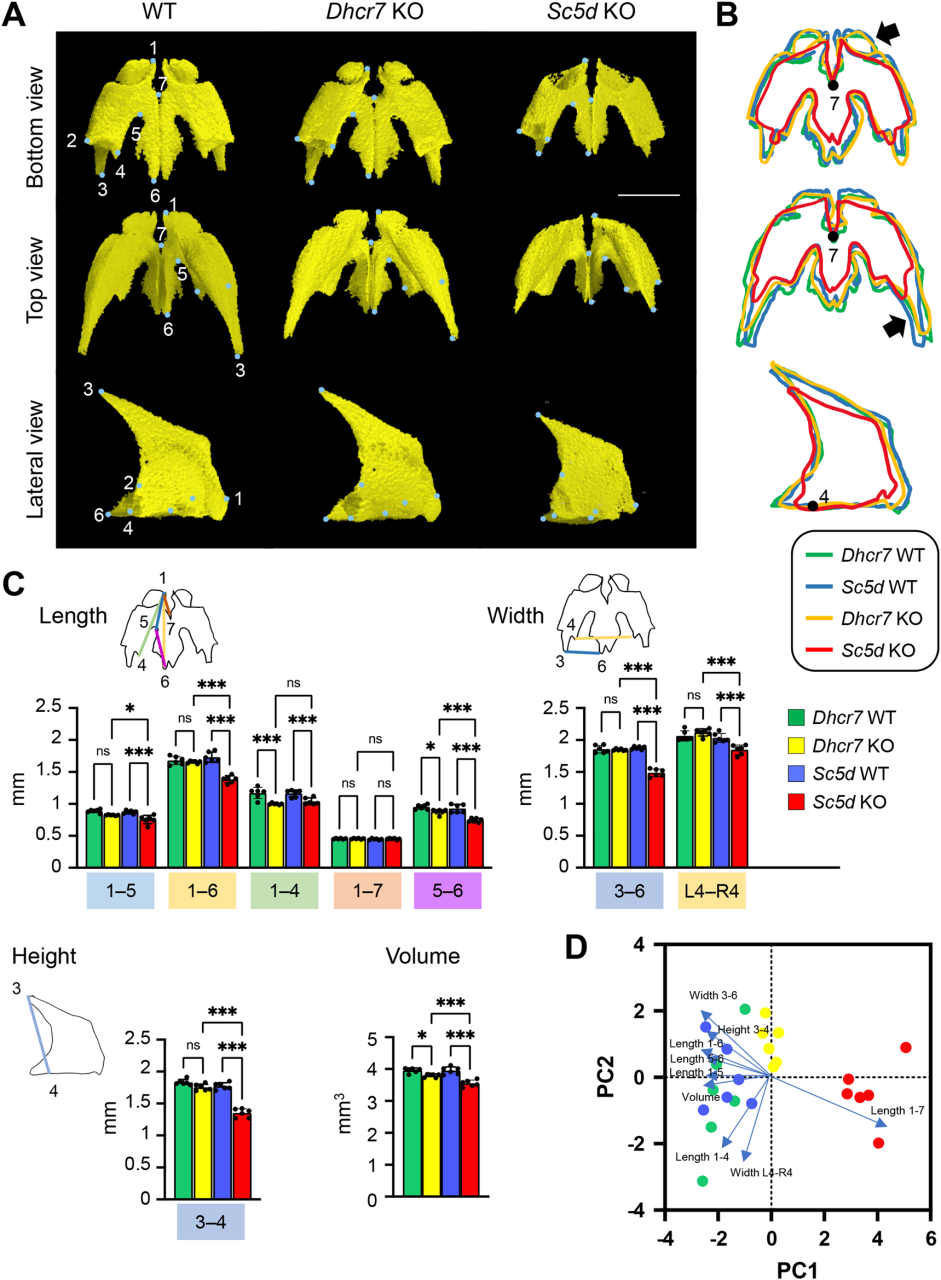
µCT analysis of the premaxilla. (**A**) 3D reconstruction of the premaxilla in E18.5 WT, *Sc5d* KO, and *Dhcr7* KO mice. Definitions of landmarks: 1. most anterior superior point of the premaxilla; 2. most lateral point of the premaxillary-maxillary suture; 3. tip of the frontal process of the premaxilla; 4. most medial point of the premaxillary-maxillary suture; 5. most anterior point of the anterior palatine foramen; 6. most posterior point of the premaxilla; and 7. most posterior point of the incisive foramen. Scale bar: 1 mm. (**B**) Wiring trace of the premaxilla in E18.5 WT (blue), *Dhcr7* KO (orange), and *Sc5d* KO (red) mice. Arrows indicate the missing portion in *Sc5d* KO mice. (**C**) Quantification of the size (length, width, height, and volume) of the maxilla from *Dhcr7* WT (green bars), *Dhcr7* KO (yellow bar), *Sc5d* WT (blue bars), and *Sc5d* KO (red bars) mice. *p < 0.05; **p < 0.01; ***p < 0.001; ns, not significant. N/A, not available. (**D**) Scatter plots of individual scores of PCA displaying the degree of morphological variances (length, width, height, and volume) of the premaxilla in *Dhcr7* WT (green dots), *Dhcr7* KO (yellow dots), *Sc5d* WT (blue dots), and *Sc5d* KO (red dots) mice, shown by PC1 and PC2. Distribution in mutants (*Sc5d* KO and *Dhcr7* KO) and control littermates (*Sc5d* WT and *Dhcr7* WT) along with 10 principal components (blue arrows) are shown.

We first defined the anatomical landmarks on the premaxilla in the 3D μCT images (Fig. 1A and Table 1). Next, to identify differences between WT controls and *Sc5d* KO and *Dhcr7* KO mice, we measured the length between landmarks, as well as total bone volume, using the 3D-reconstruction images. We found that there was no significant difference in volume in the premaxilla of *Dhcr7* KO and WT controls, whereas *Sc5d* KO mice displayed smaller premaxilla compared with either *Dhcr7* KO or WT control mice (Fig. 1B and Tables 2 and 3). In *Dhcr7* KO mice, the bone outline was almost indistinguishable from that in WT controls, although the volume was smaller than in WT controls. The palatal process of the premaxilla [length between point 5 and 6, adjusted *P* value (*P*_Adj_) = 0.035] and the body (length between point 1 and 4, *P*_Adj_ < 0.001) were statistically different between *Dhcr7* KO and WT control mice; however, total length (length between point 1 and 6) and width (length between point 3 and 6) were the same as those in WT mice (Fig. 1C and Tables 2 and 3). On the other hand, in *Sc5d* KO mice, bone volume was significantly smaller than in WT control and *Dhcr7* KO mice. Anterior–posterior lengths (between 1 and 6, and 5 and 6, respectively) and height (point 3 and 4) were significantly shorter than in WT and *Dhcr*7 KO mice. Interestingly, the most anterior part of the premaxilla (e.g. length between point 1 and 7) were indistinguishable in all genotypes (Fig. 1C, D and Table 2, and Tables S4-S6), indicating that these areas of the bone are not affected by cholesterol metabolism aberrations. Thus, in the premaxilla, *Dhcr7* KO mice displayed no major defects, whereas *Sc5d* KO exhibited hypoplastic premaxilla with major changes in its posterior part.

**Table 2:** Measurements of selected landmarks of craniofacial bones from E18.5 control, *Dhcr7* KO, and *Sc5d* KO mice.

Bone	Measurement	Landmarks	MEAN ± STDEV.P (mm)				Adjusted *P* Value			Cholesterol
			*Dhcr7* WT	*Dhcr7* KO	*Sc5d* WT	*Sc5d* KO	*Dhcr7* WT vs *Dhcr7* KO	*Dhcr7* KO vs *Sc5d* KO	*Sc5d* WT vs *Sc5d* KO	
Premaxilla	Length	1–5	0.89 ± 0.02	0.82 ± 0.01	0.87 ± 0.02	0.75 ± 0.06	0.127	0.091	< .001	Lathosterol
		1–6	1.68 ± 0.06	1.65 ± 0.02	1.73 ± 0.07	1.38 ± 0.05	0.832	< .001	< .001	Lathosterol
		1–4	1.17 ± 0.08	1 ± 0.01	1.16 ± 0.05	1.04 ± 0.05	< .001	0.521	< .001	Cholesterol
		1–7	0.45 ± 0.01	0.45 ± 0.01	0.45 ± 0.01	0.45 ± 0.01	> .999	> .999	0.997	ns
		5–6	0.95 ± 0.03	0.88 ± 0.03	0.93 ± 0.06	0.74 ± 0.03	0.035	< .001	< .001	Cholesterol
	Width	3–6	1.86 ± 0.05	1.85 ± 0.01	1.88 ± 0.02	1.49 ± 0.05	> .999	< .001	< .001	Lathosterol
		L4-R4	1.86 ± 0.09	1.79 ± 0.03	1.84 ± 0.05	1.81 ± 0.02	0.226	0.967	0.959	ns
	Height	3–4	1.83 ± 0.04	1.75 ± 0.05	1.77 ± 0.05	1.35 ± 0.06	0.081	< .001	< .001	Lathosterol
Maxilla	Length	1–3	2.55 ± 0.1	2.55 ± 0.07	2.54 ± 0.07	2.6 ± 0.1	> .999	0.609	0.324	ns
		5–10	2.38 ± 0.06	2.06 ± 0.06	2.28 ± 0.06	1.99 ± 0.07	< .001	0.239	< .001	Cholesterol
		7–8	1 ± 0.02	0.66 ± 0.02	1.01 ± 0.04	0 ± 0	< .001	< .001	< .001	Lathosterol + Cholesterol
		8–9	0.59 ± 0.01	0.35 ± 0.01	0.59 ± 0.01	0 ± 0	< .001	< .001	< .001	Lathosterol + Cholesterol
	Width	L3-R3	5.35 ± 0.13	5.23 ± 0.09	5.29 ± 0.23	5.37 ± 0.13	0.311	0.203	0.721	ns
		L5-R5	1.76 ± 0.04	1.68 ± 0.04	1.76 ± 0.03	2.37 ± 0.13	0.647	< .001	< .001	Lathosterol
		3–6	2.16 ± 0.1	2.15 ± 0.07	2.22 ± 0.05	2.1 ± 0.05	0.997	0.913	0.342	ns
		3–7	2.5 ± 0.14	2.55 ± 0.06	2.66 ± 0.19	0 ± 0	0.907	< .001	< .001	Lathosterol
	Height	1–10	1.91 ± 0.04	1.7 ± 0.04	1.89 ± 0.06	1.28 ± 0.05	< .001	< .001	< .001	Lathosterol + Cholesterol
	Distance	L6-R6	0.64 ± 0.03	0.66 ± 0.03	0.67 ± 0.01	1.12 ± 0.06	0.603	< .001	< .001	Lathosterol
		L7-R7	0.21 ± 0.01	0.24 ± 0.01	0.2 ± 0.01	0 ± 0	0.226	< .001	< .001	Lathosterol
		L8-R8	0.26 ± 0.01	0.25 ± 0.02	0.25 ± 0.02	0 ± 0	0.908	< .001	< .001	Lathosterol
		L9-R9	0.76 ± 0.03	0.74 ± 0.03	0.74 ± 0.02	0.86 ± 0.04	0.454	< .001	< .001	Lathosterol
Palatine	Length	1–4	1.76 ± 0.04	1.48 ± 0.03	1.68 ± 0.06	1.42 ± 0.07	< .001	0.24	< .001	Cholesterol
		4–7	1.48 ± 0.04	1.48 ± 0.04	1.49 ± 0.04	1.38 ± 0.05	> .999	0.017	0.007	Lathosterol
	Width	3–5	1.24 ± 0.04	1.04 ± 0.06	1.21 ± 0.03	0.84 ± 0.03	< .001	< .001	< .001	Lathosterol + Cholesterol
		L2-R2	1 ± 0.02	1.04 ± 0.03	0.97 ± 0.05	1.44 ± 0.07	0.747	< .001	< .001	Lathosterol
		L3-R3	2.43 ± 0.05	2.31 ± 0.03	2.47 ± 0.07	2.65 ± 0.03	< .001	< .001	< .001	Lathosterol
	Height	1–7	0.5 ± 0.01	0.37 ± 0.02	0.49 ± 0.03	0.24 ± 0.03	< .001	< .001	< .001	Lathosterol + Cholesterol
		3–5	0.6 ± 0.02	0.45 ± 0.04	0.6 ± 0.01	0.19 ± 0.01	< .001	< .001	< .001	Lathosterol + Cholesterol
	Distance	L4-R4	1.12 ± 0.09	1.19 ± 0.04	1.15 ± 0.06	1.22 ± 0.03	0.238	0.797	0.131	ns
		L5-R5	0.2 ± 0.01	0.25 ± 0.02	0.2 ± 0.02	0.98 ± 0.05	0.523	< .001	< .001	Lathosterol
Frontal	Length	1–2	3.39 ± 0.1	3.41 ± 0.15	3.31 ± 0.18	3.14 ± 0.06	0.994	0.014	0.192	ns
	Width	3–2	2.16 ± 0.07	2.31 ± 0.05	2.14 ± 0.09	1.5 ± 0.04	0.006	< .001	< .001	Lathosterol + Cholesterol
		3–6	2.44 ± 0.08	2.89 ± 0.07	2.35 ± 0.11	1.59 ± 0.06	< .001	< .001	< .001	Lathosterol + Cholesterol
	Height	3–4	1.4 ± 0.1	1.23 ± 0.1	1.4 ± 0.05	1.14 ± 0.09	0.034	0.612	< .001	Lathosterol
		1–4	2.66 ± 0.1	2.76 ± 0.16	2.66 ± 0.05	2.11 ± 0.07	0.493	< .001	< .001	Lathosterol
	Distance	L1-R1	0.68 ± 0.03	0.28 ± 0.02	0.65 ± 0.03	0.8 ± 0.1	< .001	< .001	< .001	Lathosterol + Cholesterol
		L2-R2	1.96 ± 0.05	1.14 ± 0.08	1.97 ± 0.07	2.52 ± 0.2	< .001	< .001	< .001	Lathosterol + Cholesterol
		L6-R6	0.57 ± 0.03	0.28 ± 0.03	0.53 ± 0.02	0.88 ± 0.09	< .001	< .001	< .001	Lathosterol + Cholesterol
Mandible	Length	1–9	5.11 ± 0.14	4.8 ± 0.07	5.29 ± 0.09	3.88 ± 0.16	< .001	< .001	< .001	Lathosterol + Cholesterol
		1–11	4.83 ± 0.11	4.38 ± 0.09	4.88 ± 0.07	3.84 ± 0.1	< .001	< .001	< .001	Lathosterol + Cholesterol
		1–15	4.61 ± 0.2	3.97 ± 0.16	4.52 ± 0.1	3.38 ± 0.13	< .001	< .001	< .001	Lathosterol + Cholesterol
		3–13	1.02 ± 0.05	0.99 ± 0.01	0.98 ± 0.02	0.92 ± 0.02	0.999	0.907	0.942	ns
	Width	L9-R9	4.83 ± 0.18	4.59 ± 0.11	4.84 ± 0.14	4.75 ± 0.1	0.006	0.136	0.601	ns
		L14-R14	0.99 ± 0.01	0.71 ± 0.06	0.95 ± 0.08	0.68 ± 0.06	< .001	> .999	0.002	7-DHC
	Height	4–14	1.5 ± 0.04	1.42 ± 0.06	1.37 ± 0.08	1.38 ± 0.05	0.28	0.842	> .999	ns
		5–12	1.32 ± 0.03	1.39 ± 0.02	1.38 ± 0.03	1.33 ± 0.03	0.157	0.31	0.48	ns
		6–11	2.22 ± 0.07	1.98 ± 0.04	2.18 ± 0.09	1.32 ± 0.01	< .001	< .001	< .001	Lathosterol + Cholesterol

**Table 3:** Comparison of the volume of craniofacial bones from E18.5 control, *Dhcr7* KO, and *Sc5d* KO mice.

Bone	Measurement	MEAN ± STDEV.P (mm3)				Adjusted *P* Value			Cholesterol
		*Dhcr7* WT	*Dhcr7* KO	*Sc5d* WT	*Sc5d* KO	*Dhcr7* WT vs *Dhcr7* KO	*Dhcr7* KO vs *Sc5d* KO	*Sc5d* WT vs *Sc5d* KO	
Premaxilla	Volume	3.96 ± 0.07	3.79 ± 0.05	3.95 ± 0.1	3.55 ± 0.09	0.022	< .001	< .001	Lathosterol
Maxilla	Volume	5.3 ± 0.1	4.95 ± 0.1	5.19 ± 0.15	4.52 ± 0.09	< .001	< .001	< .001	Cholesterol
Palatine	Volume	0.69 ± 0.03	0.63 ± 0.03	0.68 ± 0.03	0.5 ± 0.01	0.004	< .001	< .001	Lathosterol
Frontal	Volume	20.09 ± 0.6	27.62 ± 1.43	20.06 ± 1.14	14.02 ± 0.11	< .001	< .001	< .001	Lathosterol + Cholesterol
Mandible	Volume	18.13 ± 0.14	15.03 ± 0.88	17.79 ± 0.41	13.16 ± 0.07	< .001	< .001	< .001	Lathosterol + Cholesterol

### Maxilla

**Figure 2:**
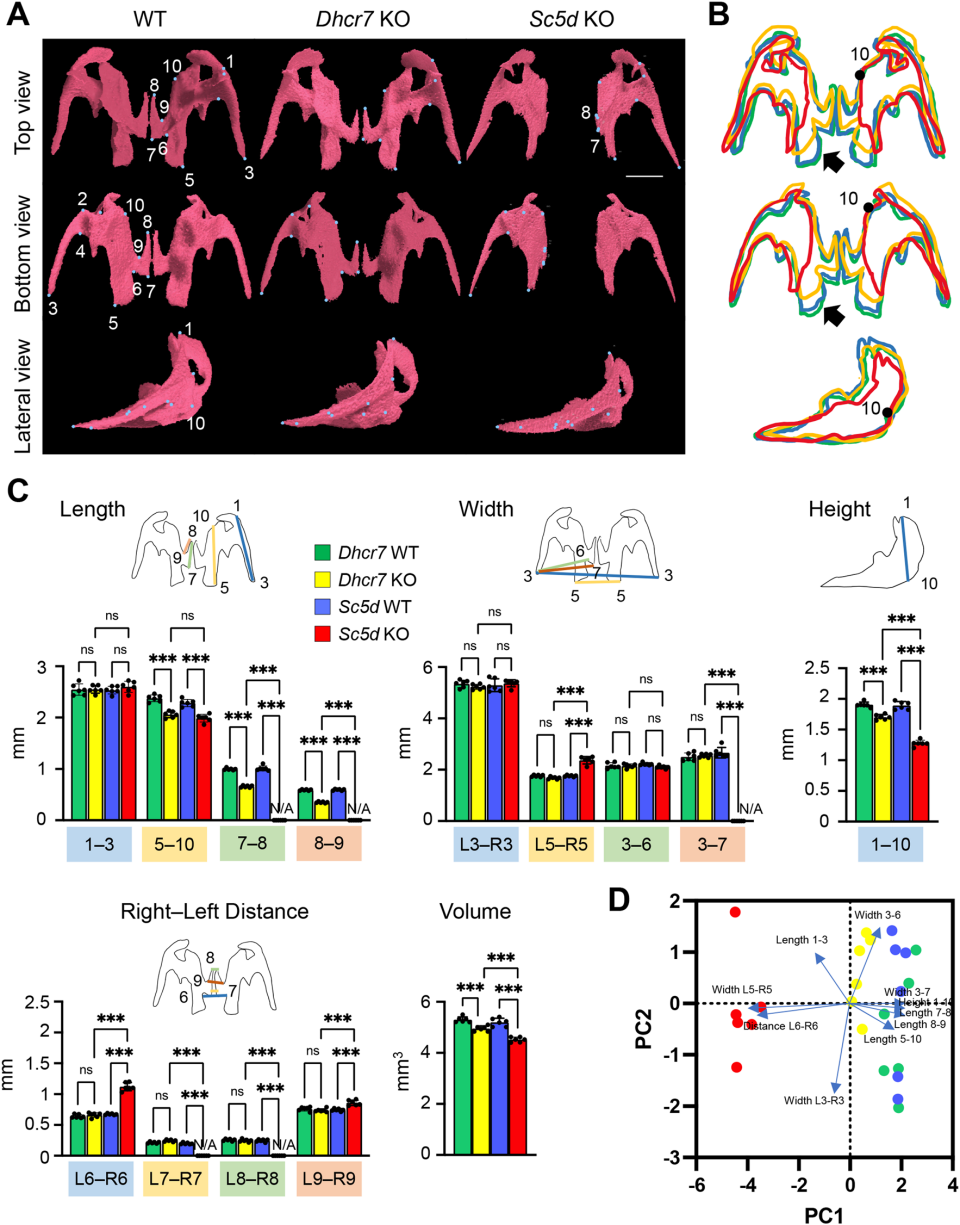
µCT analysis of the maxilla. (**A**) 3D reconstruction of the maxilla in E18.5 WT, *Dhcr7* KO, and *Sc5d* KO mice. Definitions of landmarks: 1. anterior point of the maxilla; 2. lateral inferior intersection of the frontal and zygomatic process of the maxilla; 3. tip of the zygomatic process of the maxilla; 4. anterior-medial point to the zygomatic process of the maxilla; 5. posterior point of the maxilla; 6. posterior-lateral point of the palatine process of the maxilla; 7. posterior-medial point of the palatine process of the maxilla; 8. most anterior-medial point of the palatine process of the maxilla; 9. anterior-lateral point of the palatine process of the maxilla; and 10. medial point of the premaxillary-maxillary suture. Scale bar: 1 mm. (**B**) Wiring trace of the maxilla in E18.5 WT (blue), *Dhcr7* KO (orange), and *Sc5d* KO (red) mice. Arrows indicate the missing portion in *Sc5d* KO mice. (**C**) Quantification of the size (length, width, height, right-left distance, and volume) of the maxilla from *Dhcr7* WT (green bars), *Dhcr7* KO (yellow bar), *Sc5d* WT (blue bars), and *Sc5d* KO (red bars) mice. *p < 0.05; **p < 0.01; ***p < 0.001; ns, not significant. N/A, not available. (**D**) Scatter plots of individual scores of PCA displaying the degree of morphological variances (length, width, height, right-left distance, and volume) of the maxilla in *Dhcr7* WT (green dots), *Dhcr7* KO (yellow dots), *Sc5d* WT (blue dots), and *Sc5d* KO (red dots) mice, shown by PC1 and PC2. Distributions in mutant (*Sc5d* KO and *Dhcr7* KO) and control littermate (*Sc5d* WT and *Dhcr7* WT) mice along with 14 principal components (blue arrows) are shown.

We defined the anatomical landmarks on the maxilla in the 3D μCT images (Fig. 2A and Table 1). We then measured the length between each landmark, as well as total bone volume, using the 3D-reconstruction images, and found that there were no substantial differences in the main body between *Dhcr7* KO and WT control mice (Fig. 2B). Interestingly, although there was no obvious cleft palate in *Dhcr7* KO mice, the palatal process of the maxilla (represented as the length between point 7 and 8, *P*_Adj_ < 0.001; and between 8 and 9, *P*_Adj_ < 0.001) was smaller than that in WT controls (Fig. 2C and Table 2, and Table S4). In *Sc5d* KO mice, due to cleft palate, the right-left distance between paired maxillary bones (L6‒R6 and L9‒R9) was longer compared to *Dhcr7* KO and WT control mice (Fig. 2C and Table 2, and Table S4). The distal part of the maxilla (length between point 1 and 3, *P*_Adj_ = 0.324; and between 3 and 6, *P*_Adj_ = 0.342) was not altered in *Sc5d* KO mice, whereas the palatal process of the maxilla (point 7 and 8) was missing due to cleft palate (Fig. 2C, D and Table 2, and Tables S4-S6). Interestingly, the posterior part of the maxilla was curved outward (represented by the width between left and right point 5 [L5‒R5], *P*_Adj_ < 0.001) and was longer in *Sc5d* KO mice than in WT and *Dhcr7* KO mice (Fig. 2C, D and Table 2, and Tables S4-S6). In addition, the frontal process of the maxilla (height between point 1 and 10) was significantly underdeveloped in *Sc5d* KO mice compared to *Dhcr7* KO and WT control mice (Fig. 2B and C). Overall, the volume of the maxilla was reduced in both *Dhcr7* KO and *Sc5d* KO mice, but more significantly in *Sc5d* KO mice (Fig. 2C, D and Table 3, and Tables S4-S6). Thus, *Dhcr7* KO mice showed mild bony defects in the maxilla, whereas *Sc5d* KO mice exhibited major defects in the medial part due to cleft palate.

### Palatine bone

**Figure 3:**
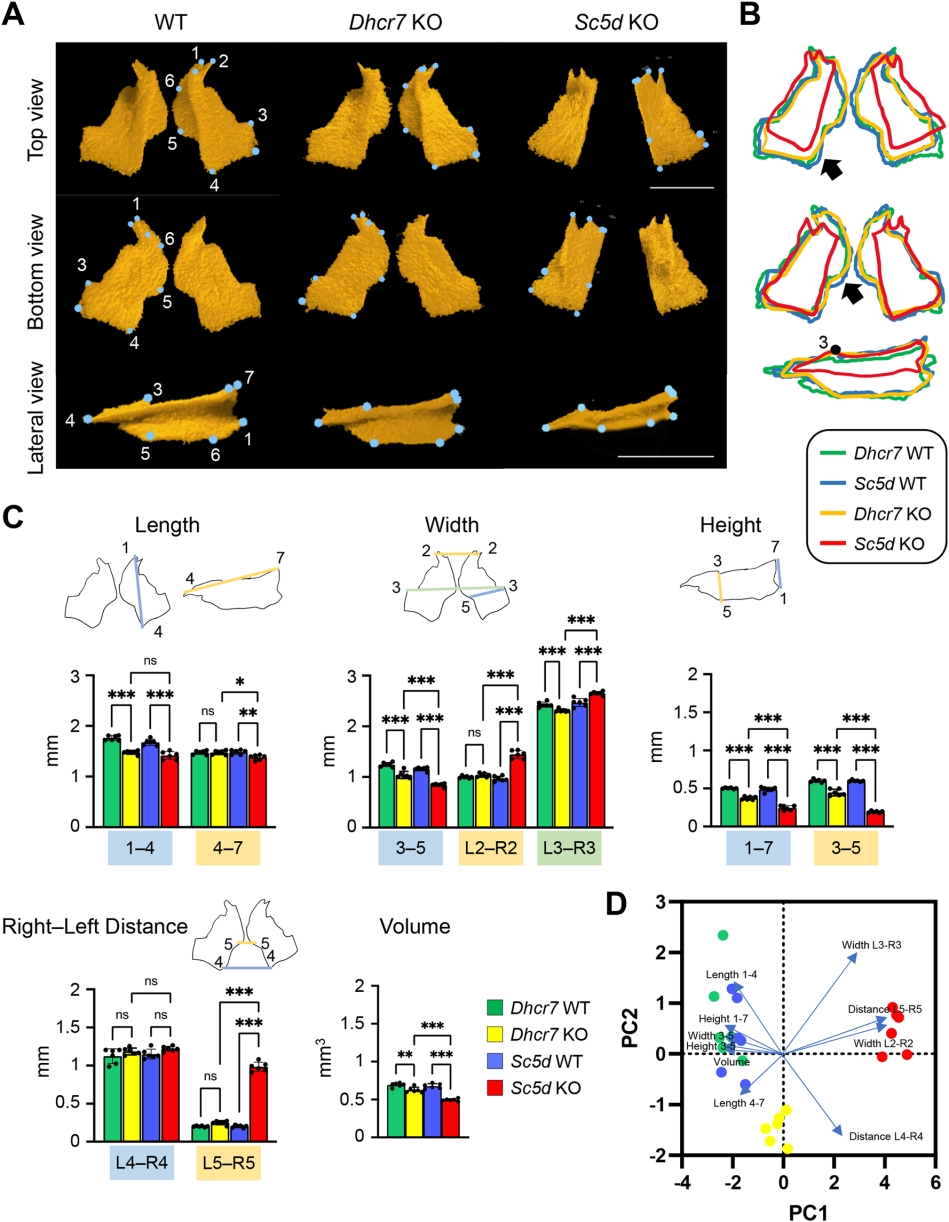
µCT analysis of the palatine bone. (**A**) 3D reconstruction of the palatine bone in E18.5 WT, *Dhcr7* KO, and*Sc5d* KO mice. Definitions of landmarks: 1. most anterior-lateral point of the palatine plate; 2. tip of the orbital process of the palatine bone; 3. lateral point of the palatine bone; 4. posterior point of the palatine bone; 5. posterior-medial point of the horizontal plate of the palatine bone; 6. anterior-medial point of the horizontal plate of the palatine bone; and 7. anterior superior point of the perpendicular plate. Scale bar: 1 mm. (**B**) Wiring trace of the palatine bone in E18.5 WT (blue), *Dhcr7* KO (orange), and *Sc5d* KO (red) mice. Arrows indicate the missing portion in *Sc5d* KO mice. (**C**) Quantification of the size (length, width, height, right-left distance, and volume) of the palatine bone from *Dhcr7* WT (green bars), *Dhcr7* KO (yellow bar), *Sc5d* WT (blue bars), and *Sc5d* KO (red bars) mice. *p < 0.05; **p < 0.01; ***p < 0.001; ns, not significant. (**D**) Scatter plots of individual scores of PCA displaying the degree of morphological variances (length, width, height, right-left distance and volume) of the palatine in *Dhcr7* WT (green dots), *Dhcr7* KO (yellow dots), *Sc5d* WT (blue dots), and *Sc5d* KO (red dots) mice, shown by PC1 and PC2. Distribution in mutants (*Sc5d* KO and *Dhcr7* KO) and control littermates (*Sc5d* WT and *Dhcr7* WT) along with 10 principal components (blue arrows) are shown.

We defined the anatomical landmarks on the palatine bone in the 3D μCT images (Fig. 3A and Table 1). Next, to identify anomalies in the palatine bone, we measured the length between landmarks, as well as total bone volume, using the 3D-reconstruction images of WT controls and *Sc5d* KO and *Dhcr7* KO mice. We found bony defects in the palatine bone in both *Dhcr7* KO and *Sc5d* KO mice, which showed reduced anterior–posterior length, medial–lateral width, and height (Fig. 3B). In agreement with our observations for the maxilla, the size of the palatine bone (represented as the length between point 1 and 4, *P*_Adj_ < 0.001; width between point 3 and 5, *P*_Adj_ < 0.001; and height between point 1 and 7, *P*_Adj_ < 0.001; and between point 3 and 5, *P*_Adj_ < 0.001) was affected in *Dhcr7* KO mice (Fig. 3C, D and Table 2, and Tables S4-S6). In *Sc5d* KO mice, the palatal process of the palatine bone was underdeveloped (point 5 and 6), resulting in cleft palate (Fig. 3C, D and Table 2, and Tables S4-S6). Interestingly, the anterior part of the palatine bones was rotated outward; therefore, the anterior part was wider than in WT controls (width between right and left point 2 [L2‒R2], *P*_Adj_ < 0.001; and between right and left point 3 [L3‒R3], *P*_Adj_ < 0.001), as seen in the posterior part of the maxilla (Figs. 2C, 3C and Table 2, and Tables S4-S6). The volume of the palatine bone was reduced in both *Dhcr7* KO (*P*_Adj_ = 0.004) and *Sc5d* KO mice (*P*_Adj_ < 0.001), but more pronouncedly in *Sc5d* KO mice (Fig. 3C, D and Table 3, and Tables S4-S6). Importantly, there was no significant difference in the anterior–posterior length of the palatine bone (length between point 1 and 4) between *Dhcr7* KO (*P*_Adj_ < 0.001) and *Sc5d* KO (*P*_Adj_ < 0.001) mice, indicating that this defect was mainly due to loss of mature cholesterol.

### Frontal bone

**Figure 4:**
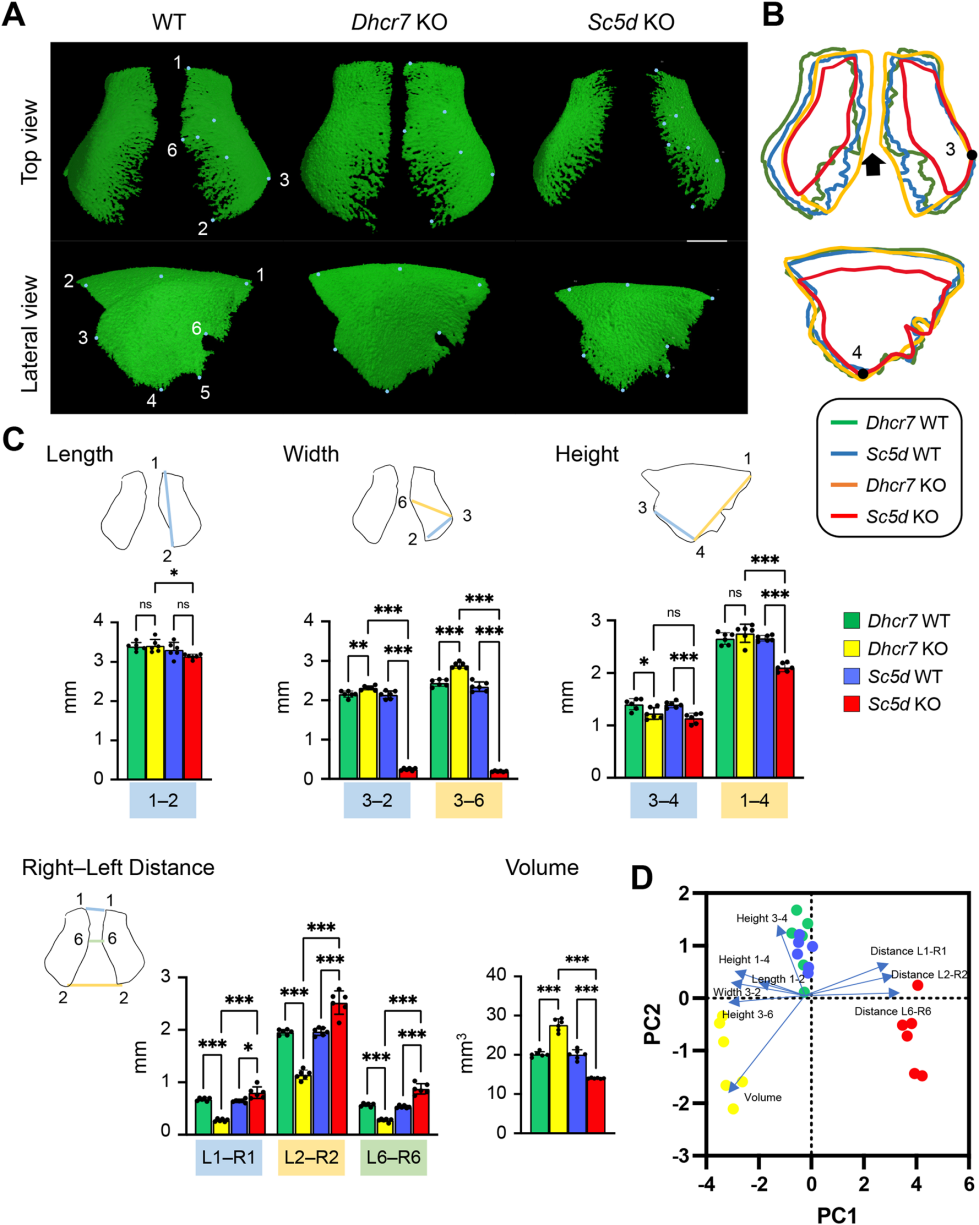
µCT analysis of the frontal bone. (**A**) 3D reconstruction of the frontal bone in E18.5 WT, *Dhcr7* KO, and *Sc5d* KO mice. Definitions of landmarks: 1. most anterior–superior point of the frontal bone; 2. most posterior-superior point of the frontal bone; 3. most posterior-lateral intersection of the frontal bone and parietal bone; 4. most posterior-inferior point of the frontal bone; 5. most anterior-inferior point of the frontal bone; and 6. midpoint of the interfrontal suture. Scale bar: 1 mm. (**B**) Wiring trace of the frontal bone in E18.5 WT (blue), *Dhcr7* KO (orange), and *Sc5d* KO (red) mice. Arrows indicate the missing portion in *Sc5d* KO mice. (**C**) Quantification of the size (length, width, height, right-left distance, and volume) of the frontal bone from *Dhcr7* WT (green bars), *Dhcr7* KO (yellow bar), *Sc5d* WT (blue bars), and *Sc5d* KO (red bars) mice. *p < 0.05; **p < 0.01; ***p < 0.001; ns, not significant. (**D**) Scatter plots of individual scores of PCA displaying the degree of morphological variances (length, width, height, right-left distance and volume) of the frontal bone in *Dhcr7* WT (green dots), *Dhcr7* KO (yellow dots), *Sc5d* WT (blue dots), and *Sc5d* KO (red dots) mice, shown by PC1 and PC2. Distributions in mutant (*Sc5d* KO and *Dhcr7* KO) and control littermate (*Sc5d* WT and *Dhcr7* WT) mice along with 9 principal components (blue arrows) are shown.

We defined the anatomical landmarks on the frontal bone in the 3D μCT images (Fig. 4A and Table 1). Next, to identify anomalies in the frontal bone, we measured the length between landmarks, as well as total bone volume, using the 3D-reconstruction images of WT controls and *Sc5d* KO and *Dhcr7* KO mice and found that bone mineralization was reduced in *Sc5d* KO mice and increased in *Dhcr7* KO mice (Fig. 4A and B). Therefore, the metopic sutures (right-left distance between point 1 [L1‒R1], *P*_Adj_ < 0.001; between point 2 [L2‒R2], *P*_Adj_ < 0.001; and between point 6 [L6‒R6], *P*_Adj_ < 0.001) were narrower in *Dhcr7* KO mice, and wider in *Sc5d* KO mice, compared to WT mice (Fig. 4C, D and Table 2, and Tables S4-S6). In agreement with these changes, the volume of the frontal bone in *Dhcr7* KO mice was increased (*P*_Adj_ < 0.001), and that in *Sc5d* KO mice was reduced (*P*_Adj_ < 0.001), compared to WT controls (Fig. 4C, D and Table 3, and Tables S4-S6). Interestingly, the anterior–posterior length of the frontal bones (length between point 1 and 2) was not changed in *Sc5d* KO (*P*_Adj_ = 0.192) and *Dhcr7* KO (*P*_Adj_ = 0.994) mice compared to WT control mice, indicating that it was not affected by any of the cholesterol metabolism aberrations (Fig. 4C, D and Table 2, and Tables S4-S6). By contrast, the height between point 3 and 4 was shorter in both *Dhcr7* KO (*P*_Adj_ = 0.034) and *Sc5d* KO (*P*_Adj_ < 0.001) mice, but no difference was observed between *Dhcr7* KO and *Sc5d* KO mice, indicating that this area of the bone was affected by loss of mature cholesterol (Fig. 4C, D and Table 2, and Tables S4-S6). Taken together, our results indicate that formation of the frontal bone may be differently regulated compared to other bones studied in this study.

### Mandible

**Figure 5:**
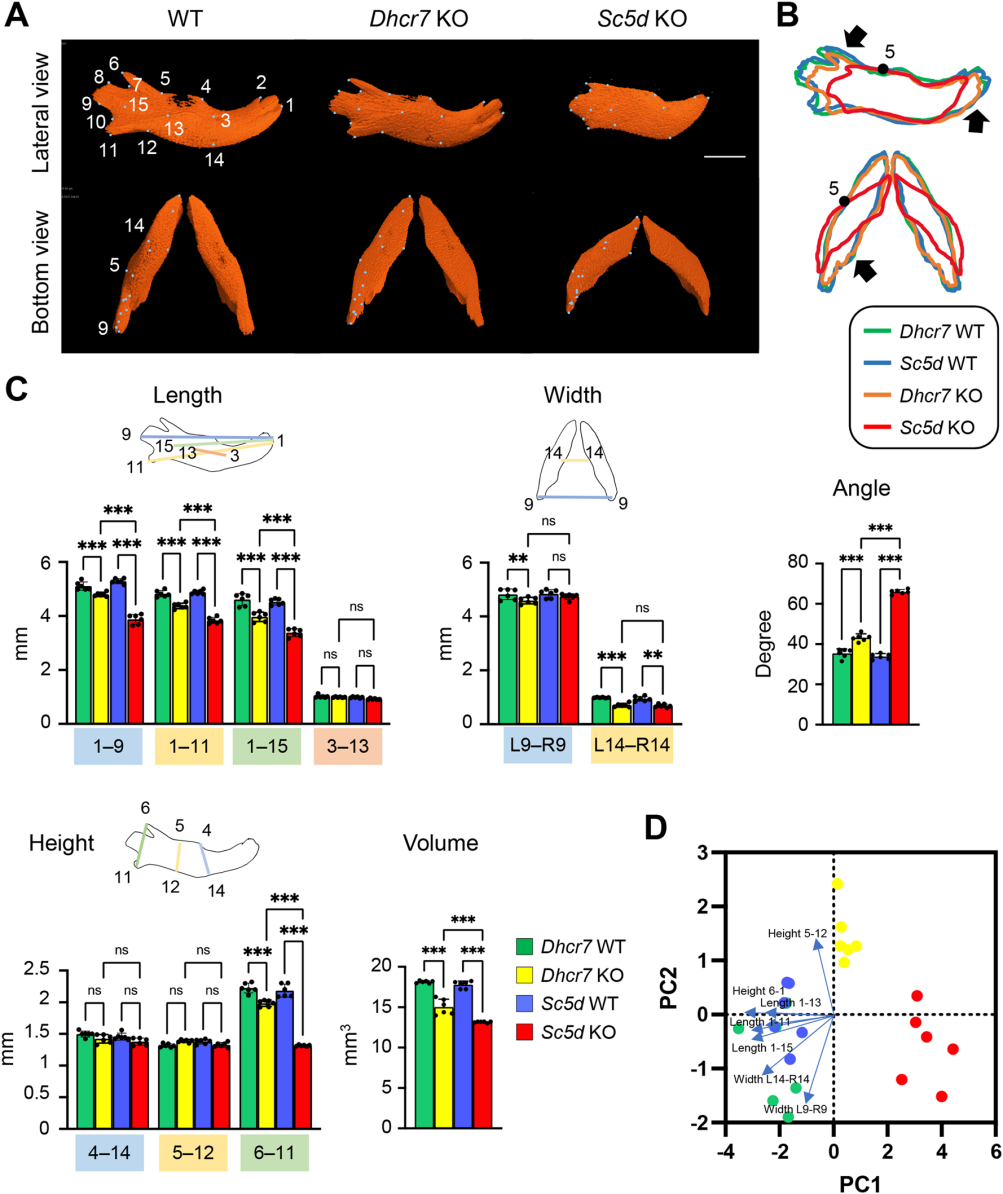
µCT analysis of the mandible. (**A**) 3D reconstruction of the mandible in E18.5 WT, *Dhcr7* KO, and *Sc5d* KO mice. Definitions of landmarks: 1. most anterior point of the mandible; 2. anterior–superior point of the mandible; 3. mental foramen; 4. molar alveolus of dentary; 5. anterior junction of the mandibular ramus and body; 6. superior tip of the coronary process of the mandible; 7. most inferior point of the mandibular notch; 8. anterior point of the condylar process of the mandible; 9. posterior point of the condylar process of the mandible; 10. superior point of the angular process of the mandible; 11. secondary cartilage of the angular process of the mandible; 12. inferior junction of the mandibular ramus and body; 13. midpoint of the external oblique ridge; 14. inferior point of the mandibular body; and 15. mandibular foramen. Scale bar: 1 mm. (**B**) Wiring trace of the mandible in E18.5 WT (blue), *Dhcr7* KO (orange), and *Sc5d* KO (red) mice. Arrows indicate the missing portion in *Sc5d* KO mice. (**C**) Quantification of the size (length, width, height, angle, and volume) of the mandible from *Dhcr7* WT (green bars), *Dhcr7* KO (yellow bar), *Sc5d* WT (blue bars), and *Sc5d* KO (red bars) mice. *p < 0.05; **p < 0.01; ***p < 0.001; ns, not significant. (**D**) Scatter plots of individual scores of PCA displaying the degree of morphological variances (length, width, height, angle, and volume) of the mandible in *Dhcr7* WT (green dots), *Dhcr7* KO (yellow dots), *Sc5d* WT (blue dots), and *Sc5d* KO (red dots) mice, shown by PC1 and PC2. Distribution in mutants (*Sc5d* KO and *Dhcr7* KO) and control littermates (*Sc5d* WT and *Dhcr7* WT) along with 11 principal components (blue arrows) are shown.

We defined the anatomical landmarks on the mandible in the 3D μCT images (Fig. 5A and Table 1). Next, to identify differences in the bone phenotype, we measured the length between landmarks, as well as total bone volume, using the 3D-reconstruction images of WT controls and *Sc5d* KO and *Dhcr7* KO mice and found that *Sc5d* KO mice displayed severe bone defects in size at the anterior and posterior parts of the mandible (Fig. 5B). We observed a missing extension along with the incisor due to mandibular incisor agenesis (point 1 and 2) and missing condylar (point 7, 8, 9, and 10), coronoid (point 5, 6, and 7), and angular (point 10 and 11) processes in the mandible (Fig. 5A and B). Interestingly, the main body of mandibular bone was not affected in both *Sc5d* KO and *Dhcr7* KO mice (anterior–posterior length between point 3 and 13, *P*_Adj_ = 0.942; height between point 4 and 14, *P*_Adj_ > 0.999; and height between point 5 and 12, *P*_Adj_ = 0.48) (Fig. 5C, D and Table 2, and Tables S4-S6). These results suggest that the extension of each process of the mandible (ramus and condylar, and coronoid processes) is highly sensitive to elevated cholesterol intermediates. The volume of the mandible was therefore decreased in both *Dhcr7* KO and *Sc5d* KO mice compared to WT controls (Fig. 5C, D and Table 3, and Tables S4-S6). Interestingly, the angle of the left and right mandible (angle R8‒1‒L8, *P*_Adj_ < 0.001) was greater in *Sc5d* KO mice compared to *Dhcr7* KO and WT control mice (Fig. 5C, D and Table 4, and Tables S4-S6), which may be due to adaptation to the widened palatine bone in *Sc5d* KO mice and/or due to independent changes caused by a shorter mandible length.

**Table 4:** Comparison of the angle of mandibles from E18.5 control, *Dhcr7* KO, and *Sc5d* KO mice.

Bone	Measurement	Landmarks	MEAN ± STDEV.P (°)				Adjusted *P* Value			Cholesterol
			*Dhcr7* WT	*Dhcr7* KO	*Sc5d* WT	*Sc5d* KO	*Dhcr7* WT vs *Dhcr7* KO	*Dhcr7* KO vs *Sc5d* KO	*Sc5d* WT vs *Sc5d* KO	
Mandible	Angle	L8-1-R8	35.4 ± 2.03	43.18 ± 1.72	33.88 ± 1.29	65.97 ± 1.02	< .001	< .001	< .001	Lathosterol + Cholesterol

## Discussion

Loss of *Dhcr7* or *Sc5d* leads to lack of mature cholesterol as well as accumulation of cholesterol intermediates, namely of 7-DHD/7-DHC or lathosterol/dehydrolathosterol, respectively. Considering the fact that phenotypes in *Dhcr7* KO mice are partially rescued with statins, which normalize the levels of cholesterol precursors^14^, craniofacial bone development is more sensitive to the accumulation of cholesterol intermediates than to absence of mature cholesterol. More recently, we have reported that mice with a deficiency for *Sc5d* exhibit mandibular hypoplasia due to defects in osteoblast differentiation^18^. *Sc5d* KO mice are smaller than littermate controls and present developmental delay and bent shorter limbs^4,18^. Interestingly, while *Sc5d* KO mice display severe defects in both endochondral and intramembranous ossification, *Dhcr7* KO mice show less severe bone defects^14,18^. The phenotypic differences between *Dhcr7* KO and *Sc5d* KO mice may depend on the expression pattern of *Dhcr7* and *Sc5d*. In addition, molecules that are modified with cholesterol may be differentially expressed at each bone and location during craniofacial development. For instance, hedgehog ligands (Sonic Hedgehog [SHH], Indian Hedgehog [IHH], and Desert Hedgehog [DHH]) and receptor Smoothened (SMO) are known to be modified with cholesterol, which is crucial for their distribution and activity^21,22^. For example, an absent cholesterol modification on SHH (ShhN) leads to a shorter distribution and lower activation of SHH signaling in limb buds, but no difference in its biological functions compared to the cholesterol-modified molecules^23,24^. Although oxysterols have a variety of biological activities and affect cell survival, apoptosis, gene expression, as well as Hedgehog signaling activity, it remains unknown to what extent oxysterols derived from 7-DHC, lathosterol, and cholesterol differ in cell toxicity^25,26^. In addition, the plasma membrane contains cholesterol-rich micro-domains (e.g. lipid rafts and caveolae) that act as a signaling center by assembling receptors and channels^27,28^, and transduction and activation of hedgehog signaling is regulated by oxysterols and binding of cholesterol on the membrane^29–31^. Therefore, a precisely controlled cholesterol synthesis process is important for cellular functions. Our results show that bone formation was differently affected in each mutant mouse model analyzed, indicating a location-specific requirement and the role of cholesterol metabolism in bone development.

This study shows how bone formation (e.g. size and volume) is affected in each craniofacial bone from *Dhcr7* KO *and Sc5d* KO mice. We found that loss of mature cholesterol had a lower impact on bone formation than elevated levels of cholesterol intermediates. In the mandible, the extension of the process of the mandible was drastically affected in *Sc5d* KO mice compared to *Dhcr7* KO and WT control mice. Because these areas are formed through endochondral ossification, chondrocytes may be more sensitive to elevated cholesterol intermediates compared to osteoblasts. Future studies may identify specific functions for each cholesterol intermediate in various cell types.

One of the limitations of this study is that there are some differences in the phenotypes observed in humans and mice. For instance, while *DHCR7* mutations in humans causes SLOS with growth retardation, microcephaly, micrognathia, and cleft palate, *Dhcr7* KO mice display a distinct cleft palate with less than 10% penetrance^16^. It should be noted that, in this study, we analyzed *Dhcr7* KO mice without cleft palate. In addition, while a larger volume for the frontal bone was seen in *Dhcr7* KO mice compared to WT littermates, *Dhcr7* KO mice displayed smaller skulls at birth^14^. The accelerated bone formation and differentiation observed in *Dhcr7* KO mice^14^ might lead to immature closure of sutures between bones as well as growth arrest in the growth plate of long bones. In humans, children with SLOS typically show a head circumference 2 standard deviations below the average of unaffected children. Although no three dimensional volumetric analysis was conducted in this study, overall craniofacial features seem to be conserved in *Dhcr7* KO mice. Thus, the findings in mouse models need to be further evaluated in SLOS patients.

### Supplementary Information

Supplementary Figure S1.

Supplementary Tables.

## Data Availability

All data needed to evaluate the conclusions in the paper are present in the article and/or the supplemental materials. Additional data related to this paper are available from the corresponding author upon reasonable request.
