# Supplementary material for: Craniofacial bone anomalies related to cholesterol synthesis defects
Source: Sci Rep. 2024 Mar 4;14:5371. doi: 10.1038/s41598-024-55998-3 (PMC10912708; doi:10.1038/s41598-024-55998-3)

Supplemental Information

Figure S1. μCT analysis of the premaxilla, maxilla, palatine bone, frontal bone, and mandible in WT littermates of *Sc5d* KO and *Dhcr7* KO mice. 3D reconstruction of the premaxilla (yellow), maxilla (pink), palatine bone (orange), frontal bone (green), and mandible (red) in E18.5 *Sc5d* WT and *Dhcr7* WT mice. Scale bar: 1 mm.

Figure S1. Iwaya et al.

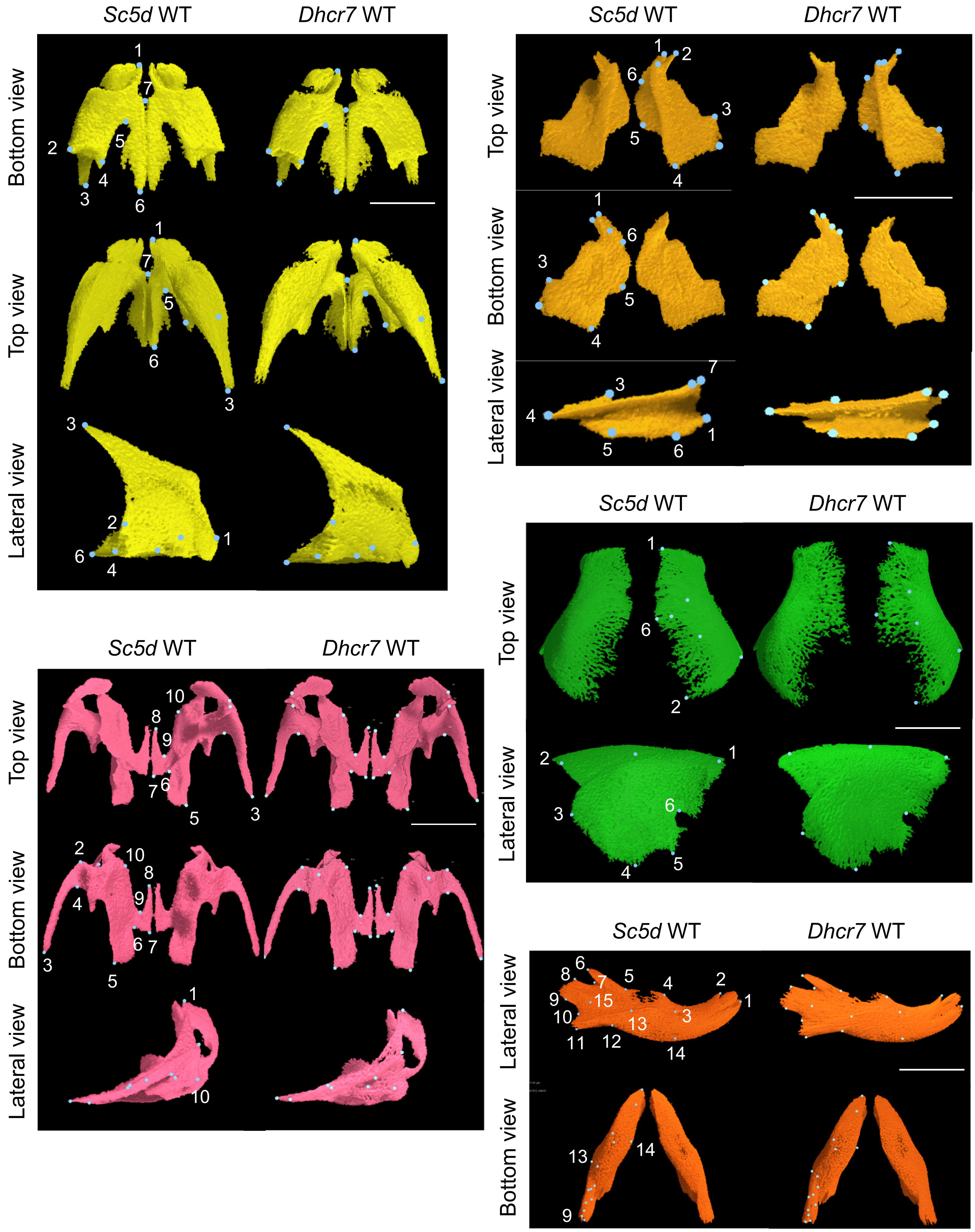

Supplement: Supplementary file 1 — Supplementary Figure S1. [file 41598_2024_55998_MOESM1_ESM.pdf]
